# Comparative chloroplast genome analysis of *Ficus* (Moraceae): Insight into adaptive evolution and mutational hotspot regions

**DOI:** 10.3389/fpls.2022.965335

**Published:** 2022-09-15

**Authors:** Zheng-Ren Zhang, Xue Yang, Wei-Ying Li, Yan-Qiong Peng, Jie Gao

**Affiliations:** ^1^CAS Key Laboratory of Tropical Forest Ecology, Xishuangbanna Tropical Botanical Garden, Chinese Academy of Sciences, Mengla, China; ^2^University of Chinese Academy of Sciences, Beijing, China; ^3^College of Life Sciences, Jilin Agricultural University, Changchun, China; ^4^Southwest Research Center for Landscape Architecture Engineering Technology, State Forestry and Grassland Administration, Southwest Forestry University, Kunming, China

**Keywords:** *Ficus*, chloroplast genome, nucleotide diversity hotspots, phylogenetic relationship, adaptive evolution, divergence time

## Abstract

As the largest genus in Moraceae, *Ficus* is widely distributed across tropical and subtropical regions and exhibits a high degree of adaptability to different environments. At present, however, the phylogenetic relationships of this genus are not well resolved, and chloroplast evolution in *Ficus* remains poorly understood. Here, we sequenced, assembled, and annotated the chloroplast genomes of 10 species of *Ficus*, downloaded and assembled 13 additional species based on next-generation sequencing data, and compared them to 46 previously published chloroplast genomes. We found a highly conserved genomic structure across the genus, with plastid genome sizes ranging from 159,929 bp (*Ficus langkokensis*) to 160,657 bp (*Ficus religiosa*). Most chloroplasts encoded 113 unique genes, including a set of 78 protein-coding genes, 30 transfer RNA (tRNA) genes, four ribosomal RNA (rRNA) genes, and one pseudogene (*infA*). The number of simple sequence repeats (SSRs) ranged from 67 (*Ficus sagittata*) to 89 (*Ficus microdictya*) and generally increased linearly with plastid size. Among the plastomes, comparative analysis revealed eight intergenic spacers that were hotspot regions for divergence. Additionally, the *clpP*, *rbcL*, and *ccsA* genes showed evidence of positive selection. Phylogenetic analysis indicated that none of the six traditionally recognized subgenera of *Ficus* were monophyletic. Divergence time analysis based on the complete chloroplast genome sequences showed that *Ficus* species diverged rapidly during the early to middle Miocene. This research provides basic resources for further evolutionary studies of *Ficus*.

## Introduction

Chloroplasts originate from the endosymbiotic relationship between free-living cyanobacteria and eukaryotic cells (Wicke et al., 2011) and are responsible for photosynthesis, which is crucial for plant adaptation and evolution (Yin et al., 2018; Gao et al., 2019b; Thode and Lohmann, 2019). In angiosperms, chloroplast genomes typically exhibit a quadripartite structure, including a pair of inverted repeat (IR) regions, a large single-copy (LSC) region, and a small single-copy (SSC) region (Shinozaki et al., 1986; Abdullah et al., 2021). The number of unique genes generally ranges from 110 to 130 (Green, 2011; Chen et al., 2015; Hu et al., 2016). Furthermore, compared to the nuclear genome, the maternally inherited chloroplast genome is small and haploid, with no recombination and low mutation. Thus, it is highly conserved in structure and gene composition. Based on these characteristics, chloroplast genome sequence data should provide robust solutions for studies on phylogenetics, molecular evolution, population genetics, and phylogeography (Dong et al., 2013; Yang et al., 2020, 2021b; Du et al., 2021).

With the rapid development of DNA barcode identification technology, chloroplast markers have been widely used in inter- and intraspecific analysis of various plant species and populations (Riggins and Seigler, 2012; Li et al., 2015; Wu et al., 2021). For example, the *matK* and *trnH-psbA* datasets can identify more than 94% of species in Myristicaceae (Newmaster et al., 2008), and five widely used gene regions (*rbcL*, *matK*, *rpoC1*, *trnH-psbA*, and *atpF-atpH*) can correctly identify more than 97% of Canadian temperate flora samples (Burgess et al., 2011). However, none of these Sanger-sequenced chloroplast markers are universal for all plant taxa, and they provide limited information at the subspecies level (Yang et al., 2018; Huang et al., 2021b). In contrast, whole chloroplast genomes provide more extensive and higher resolution information (Wei et al., 2017; Luo et al., 2021).

In plants, chloroplasts not only perform photosynthesis but also play pivotal roles in carbohydrate, protein, and lipid biosynthesis, thus impacting growth and development (Jensen and Leister, 2014). As such, an increasing number of studies have shown that certain chloroplast genes are or have been under positive selection, yielding adaptive changes across taxa (Yang et al., 2005, 2020). For example, to better understand sunlight preferences in *Oryza*, Gao et al. (2019b) identified 14 chloroplast genes under positive selection in response to diverse habitats. Huang et al. (2021b) and Liu et al. (2021) also identified several positively selected chloroplast genes (e.g., *matK*, *ycf*2, *accD*, *clpP*, *petA*, *rps13*, and *rpoC2*) involved in adaptation to sunlight intensity in white oak and *Bupleurum*, respectively. Yao et al. (2019) identified a positively selected gene (*rbcL*) in *Ilex* associated with wet and dry habitats, contributing to the adaptation of introgressed individuals to changing environments. Hence, natural selection on the chloroplast genome influences plant adaptation through its evolutionary history (Wu et al., 2020).

*Ficus* (Moraceae) contains approximately 850 species (Shanahan et al., 2001) divided into six subgenera (*Sycomorus*, *Synoecia*, *Urostigma*, *Pharmacosycea*, *Ficus*, and *Sycidium*). *Ficus* species are widely distributed across tropical and subtropical regions (Berg and Corner, 2005) and provide essential food resources (infructescences) for frugivorous animals, making them keystone species in tropical forest ecosystems (Dev et al., 2011). *Ficus* species also exhibit significant coevolutionary relationships with obligate mutualist pollinating wasps (Cruaud et al., 2012). Over the past 25 years, phylogenetic studies on *Ficus* have been conducted based on nuclear markers [e.g., internal transcribed spacer (ITS), external transcribed spacer (ETS), glyceraldehyde 3-phosphate dehydrogenase (*G3pdh*), chloroplast expressed glutamine synthetase region (*ncpGS*), and granule-bound starch synthase (waxy region)] and chloroplast genes (e.g., *rbcL* and tRNA spacers). However, these studies have been unable to confidently resolve relationships among major groups of figs and have revealed conflicts with traditional morphological classifications (Herre et al., 1996; Weiblen, 2000; Jousselin et al., 2003; Ronsted et al., 2005, 2008; Xu et al., 2011; Cruaud et al., 2012). Bruun-Lund et al. (2017) constructed the first whole chloroplast genome-based phylogenetic tree for figs and detected a high level of cyto-nuclear discordance in several subgenera.

An unambiguously resolved *Ficus* phylogeny will facilitate key research on diversification, biogeography, and species interactions, and could be achieved using whole chloroplast genomes. Furthermore, comparative analysis of whole chloroplast genomes may provide an accurate and rapid method to discriminate species and subspecies (Li et al., 2015). This contrasts with the use of limited chloroplast regions, which exhibit low resolution in identifying the phylogeographic structure of geographically widespread figs (Huang et al., 2021a) and an inability to differentiate closely related species, such as those within the *Ficus auriculata* complex, due to high sequence similarity caused by rapid radiation and hybridization (Wu et al., 2003; Berg and Corner, 2005; Zhang et al., 2018, 2019). Whole chloroplast genome sequencing may help reveal divergent hotpot regions to address these and other unanswered questions in *Ficus*. Furthermore, *Ficus* species exhibit considerable life-form diversity, including trees, shrubs, stranglers, epiphytes, hemi-epiphytes, climbers, lithophytes, and rheophytes (Harrison et al., 2012; Bruun-Lund et al., 2017; Machado et al., 2018; Clement et al., 2020; Zhang et al., 2020b), and can survive in a variety of ecological niches (Harrison, 2005), indicating that different figs have adapted to diverse habitats. Therefore, chloroplast genomes may exhibit high levels of variation among species due to differing adaptations. Although the availability of sequenced *Ficus* chloroplast genomes has increased in recent years (Bruun-Lund et al., 2017; Liu et al., 2019; Wang et al., 2021; Lin et al., 2022), large-scale comparative analysis across all six subgenera has not yet been conducted. Therefore, the evolution of *Ficus* chloroplasts remains poorly understood.

In this study, we sequenced and assembled 13 whole chloroplast genomes from 10 *Ficus* species. In addition, we assembled 21 plastomes from 13 *Ficus* species using publicly available next-generation sequencing data. We also downloaded six previously reported *Ficus* plastomes from the NCBI database. Overall, we obtained the complete chloroplast genomes of 24 *Ficus* species covering all six subgenera. The aims of this study were to: (1) reveal chloroplast genomic variation across the genus and screen for hypervariable regions among species; (2) identify the adaptive evolution of protein-coding plastid genes within *Ficus*; and (3) construct phylogenetic trees and estimate divergence times for *Ficus* based on the whole chloroplast genomes and 40 plastomes of *Ficus* species obtained from previous research. The results of this study should improve our understanding of chloroplast genome evolution and its contribution to the evolutionary and ecological success of this economically and evolutionarily important genus.

## Materials and methods

### Genomic DNA extraction, sequencing, assembly, and annotation

We collected 13 individuals representing 10 species of *Ficus* [*F. auriculata*, *F. oligodon*, *F. hainanensis*, *F. beipeiensis*, *F. religiosa*, *F. tinctoria*, *F. ischnopoda*, *F. langkokensis*, *F. pumila*, and newly identified lineage *F. northern* (*Fn*) (Gao et al., unpublished data)] from Xishuangbanna (southern Yunnan), Gengma (northwestern Yunnan), and Chongqing, China. All voucher specimens have been preserved in the Lab of Coevolution Research Group in Xishuangbanna Tropical Botanical Garden, Xishuangbanna, China. We also obtained whole genomic DNA data of 21 samples and two outgroup species (*Antiaris toxicaria* and *Morus alba*; both Moraceae) from the BIG Data Center^1^ under BioProject accession number GSA: PRJCA002187 (Zhang et al., 2020b) and six chloroplast genomes from six species of figs from the NCBI database. In total, we obtained samples from 42 individuals representing 40 accessions and 24 species of *Ficus*, including at least two species from each of the six subgenera (Supplementary Table 1), and two outgroup taxa.

For the 13 newly sequenced figs, we collected fresh clean leaves and stored them in liquid nitrogen until further processing. From these samples, we extracted genomic DNA using a DNAsecure Plant kit (TIANGEN, Beijing, China). The purified DNA was then used to generate 350-bp fragments for library construction using paired-end sequencing on the Illumina HiSeq 2500 platform (Illumina Inc., San Diego, CA, United States). For the newly sequenced plastomes and downloaded genomes (34 samples in total), we performed *de novo* assembly using GetOrganelle v1.7.1 (Jin et al., 2020), and checked the generated circular plastid graphs using Bandage v0.8.1 (Wick et al., 2015). We used *F. auriculata* (accession number: OK078618) as a reference for CPGAVAS2 to automatically annotate the plastome of *F. hainanensis* (FSP2) and conducted manual adjustments using Geneious Prime 2020.0.5 (Kearse et al., 2012). Thereafter, we manually annotated the remaining 41 plastomes and compared them with the reference sequence FSP2. We deposited the newly generated plastid genome sequences in GenBank (accession numbers ON711000–ON711012) and used OGDRAW^2^ (Greiner et al., 2019) to visualize the circular genome maps of the *Ficus* species.

### Sequence comparison and simple sequence repeat analyses

We used the mVISTA online tool^3^ (Frazer et al., 2004) to analyze plastome divergence of all 24 species of *Ficus*, with *F. auriculata* (BN-FA10) as a reference. We also aligned the complete chloroplast genome sequences in MAFFT v7.313 using default parameters (Katoh and Standley, 2013) and calculated nucleotide diversity (π) in DnaSP v5.10.01 (Librado and Rozas, 2009) with a window length of 600 bp and step size of 200 bp. We implemented MISA-web^4^ (Beier et al., 2017) to identify simple sequence repeats (SSRs) in the plastomes of the 24 species. Minimal repeat numbers were set as mononucleotides ≥ 10, dinucleotides ≥ 5, trinucleotides ≥ 4, tetranucleotides ≥ 4, pentanucleotides ≥ 4, and hexanucleotides ≥ 4. Furthermore, we calculated the correlation between chloroplast sequence length and number of SSRs using R (R Core Team, 2013). Spearman rank correlation was used to assess *p*-values.

### Phylogenetic analyses and molecular dating

To gain insight into the phylogeny of figs, we downloaded the chloroplast sequences of individual IR, SSC, and LSC regions of 40 other fig species (Bruun-Lund et al., 2017), and performed phylogenetic analysis of a total of 63 fig species, with two Moraceae species used as the outgroup. All sequences with one IR were aligned with MAFFT v7.313, after which all indels were removed using trimAI (Capella-Gutierrez et al., 2009). Phylogenomic analyses were conducted using maximum-likelihood (ML) and Bayesian inference (BI). The ML tree was reconstructed using IQ-TREE (Nguyen et al., 2015) based on the best-fit nucleotide substitution model selected by ModelFinder (Kalyaanamoorthy et al., 2017). Branch support for the ML tree was evaluated by 10,000 ultrafast bootstrap replicates (Minh et al., 2013). MrBayes 3.2.6 (Ronquist et al., 2012) was used for the BI tree, with 2,000,000 generations under the GTR + F + I + G4 model with three heated chains and one cold chain, where the initial 25% of sampled data were discarded as burn-in. At the end of the run, average standard deviation of split frequencies was < 0.01 and effective sample size (ESS) was larger than 200. All procedures were completed in PhyloSuite v1.2.2 (Zhang et al., 2020a).

We estimated divergence times using BEAST v1.10.4 (Drummond and Rambaut, 2007) with an uncorrelated lognormal relaxed clock. For the tree prior, we applied the Yule and Birth-Death speciation process. The crown divergence time of *Ficus* [74.9 million years ago (Mya)] and Moraceae (93.1 Mya) was constrained by secondary calibrations using normal distribution based on previous studies (Cruaud et al., 2012; Gardner et al., 2017). We ran Markov Chain Monte Carlo (MCMC) for 800 million generations with sampling every 1,000 cycles. The first 10% of samples were discarded as burn-in. We examined the outputs using Tracer v1.7 (Rambaut et al., 2018) to confirm convergence based on ESS > 200.

To explore the relationship between fig and wasp phylogenies, we directly compared our ML tree of *Ficus* with a pollinating wasp phylogeny from previous analysis (Cruaud et al., 2012).

### Genome-wide scan for positively selected genes

We first calculated the non-synonymous rate (*dN*), synonymous rate (*dS*), and substitution ratio (ω = *dN*/*dS*) of all 78 chloroplast protein-coding genes using EasyCodeML v.1.31 (Gao et al., 2019a). Purifying selection, neutral selection, and positive selection were interpreted as ω < 1, ω = 1, and ω > 1, respectively.

We extracted each protein-coding gene in Geneious Prime 2020.0.5 and aligned them using MAFFT v7.313. Before calculation, all gaps and stop codons were removed, and multiple aligned genes with similar functions were concatenated into a single matrix, e.g., 11 genes (*ndhJ*, *ndhK*, *ndhC*, *ndhB*, *ndhH*, *ndhA*, *ndhI*, *ndhG*, *ndhE*, *ndhD*, and *ndhF*) related to NADH oxidoreductase were concatenated. In total, 17 matrices were obtained, including *accD*, *atp*, *ccsA*, *cemA*, *clpP*, *matK*, *ndh*, *pet*, *psa*, *psb*, *rbcL*, *rpl*, *rpo*, *rps*, *ycf1*, *ycf2*, and *ycf3* plus *ycf4*. The ML tree was constructed using the above methods (plastome sequences with single IR, SSC, and LSC regions) to detect the selection. Based on the ML tree, we performed selective pressure analysis using branch, site, and branch-site models with the CODEML algorithm in EasyCodeML v.1.31 to estimate ω. Branch models were used to detect different branches in the tree showing significant differences in ω (Yang and Nielsen, 1998, 2002). Two branch models were used: i.e., one-ratio model (M0) assuming the same ω for all branches in the tree, and two-ratio model assuming foreground branches have a specific ω that varies from background branches (Yang, 1998). Site models were used to locate positively selected sites in aligned sequences (Yang and Nielsen, 2002), assuming that ω is the same for all branches in the phylogenetic tree but varies among sites in aligned sequences. Two site models were used: i.e., M1a (nearly neutral) and M2a (positive selection) (Gao et al., 2019a). In the branch-site model, Model A_*null*_ assumes that the foreground branch is not under positive selection; conversely, Model A allows positive selection in the foreground branch (Zhang et al., 2005). We performed three pairwise comparisons (i.e., M0/two-ratio model; M1a/M2a; Model A_*null*_/Model A) using likelihood ratio tests to check for significance. In addition, we analyzed the *dN*, *dS*, and *dN*/*dS* values of all protein-coding gene datasets among the 24 representative fig species with TBtools (Chen et al., 2020).

## Results

### Structure and features of *Ficus* plastomes

**FIGURE 1:**
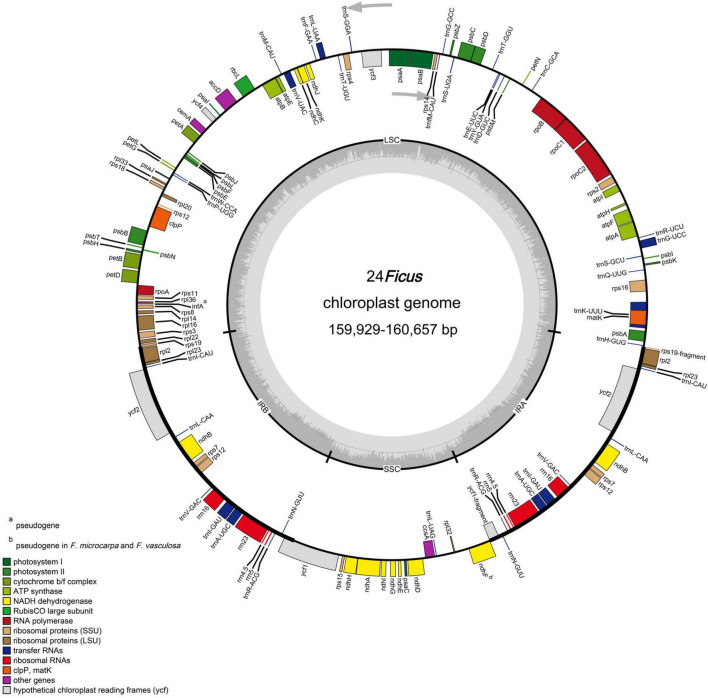
Representative chloroplast genome of *Ficus*. Genes shown on the outside of the circle are transcribed counter-clockwise, and genes inside are transcribed clockwise. Genes belonging to different functional groups are color-coded. The darker gray in the inner corresponds to GC content, and the lighter gray corresponds to AT content.

We obtained the plastomes of 24 species of *Ficus*. The complete chloroplast genomes ranged in size from 159,929 bp (*F. langkokensis*) to 160,657 bp (*F*. *religiosa*) and displayed a typical quadripartite structure, including a pair of IR regions (IRa and IRb) ranging from 25,830 bp in *F. religiosa* (W62) to 25,912 bp in *F. adhatodifolia* and *F. langkokensis*, a LSC region ranging from 88,215 bp in *F. langkokensis* to 88,873 bp in *F. religiosa* (WG62), and a SSC region ranging from 19,871 bp in *F. maxima* to 20,165 bp in *F. erecta* (Figure 1 and Supplementary Table 2). GC content ranged from 35.9–36.1%, 33.4–33.7%, 28.9–29.1%, and 42.6–42.7% in the whole chloroplast genomes and LSC, SSC, and IR regions, respectively (Supplementary Table 2).

The chloroplast genomes each encoded 113 unique genes, consisting of 78 protein-coding genes, 30 transfer RNA (tRNA) genes, four ribosomal RNA (rRNA) genes, and one pseudogene (*infA*) (Table 1). In addition, *ndhF* was identified as a pseudogene in *F. microcarpa* and *F. vasculosa*. In total, 17 genes were duplicated in IR regions, including six protein-coding genes, seven tRNAs, and all four rRNAs (Table 1). Among them, 15 contained one intron (*rpl2*, *ndhB*, *trnI-GAU*, *trnA-UGC*, *ndhA*, *rpl16*, *petD*, *petB*, *trnV-UAC*, *trnL-UAA*, *rpoC1*, *atpF*, *trnG-UCC*, *rps16*, and *trnK-UUU*) and three contained two introns (*ycf3*, *clpP*, and *rps12*). In addition, *rps12* was *trans-*spliced with two exons, with a 5′ exon in the LSC region and a 3′ exon in the IR region. In all 24 species of *Ficus*, the *rps19* and *ycf1* genes crossed the LSC/IRb and SSC/IRb junctions, respectively, and their fragments were present at the IRa/LSC and IRa/SSC borders, respectively. Furthermore, *ndhF* was traversed at the SSC/IRa junction and *rpl2* and *trnH-GUG* flanked the IRa/LSC junction (Figure 1).

**TABLE 1:** Gene composition of *Ficus* chloroplast genomes.

Category	Group of genes	Genes
Photosynthesis related genes	Photosystem I	*psaB, psaA, psaI, psaJ, psaC*
	Photosystem II	*psbA, psbK, psbI, psbM, psbD, psbC, psbZ, psbJ, psbL, psbF, psbE, psbB, psbT, psbN, psbH*
	Cytochrome b6/f complex	*petN, petA, petL, petG, petB*^a^*, petD*^a^**
	ATP synthase	*atpA, atpF*^a^*, atpH, atpI, atpE, atpB*
	Rubisco	*rbcL*
	Assembly/stability	*ycf3*^b^*, ycf4*
	NADH oxidoreductase	*ndhJ, ndhK, ndhC, ndhB*^ac^*, ndhH, ndhA*^a^*, ndhI, ndhG, ndhE, ndhD, ndhF*
	Cytochrome c synthesis	*ccsA*
Transcription and translation related genes	Large subunit ribosomal proteins	*rpl33, rpl20, rpl36, rpl14, rpl16*^a^*, rpl22, rpl2*^ac^*, rpl23*^c^*, rpl32*
	Small subunit ribosomal proteins	*rps16*^a^*, rps2, rps14, prs4, rps18, rps12*^bc^*, rps11, rps8, rps3, rps19, rps7*^c^*, rps15*
	Transcription	*rpoC2, rpoC1*^a^*, rpoB, rpoA*
	Translation initiation factor	*infA^d^*
	Ribosomal RNAs	*rrn16*^c^*, rrn23*^c^*, rrn4.5*^c^*, rrn5*^c^**
	Transfer RNAs	*trnH-GUG, trnK-UUU*^a^*, trnQ-UUG, trnS-GCU, trnG-UCC*^a^*, trnR-UCU, trnC-GCA, trnD-GUC, trnY-GUA, trnE-UUC, trnT-GGU, trnS-UGA, trnG-GCC, trnfM-CAU, trnS-GGA, trnT-UGU, trnL-UAA*^a^*, trnF-GAA, trnV-UAC*^a^*, trnM-CAU, trnW-CCA, trnP-UGG, trnI-CAU*^c^*, trnL-CAA*^c^*, trnV-GAC*^c^*, trnI-GAU*^ac^*, trnA-UGC*^ac^*, trnR-ACG*^c^*, trnN-GUU*^c^*, trnL-UAG*
Other genes	Carbon metabolism	*cemA*
	RNA processing	*matK*
	Proteolysis	*clpP^b^*
	Fatty acid synthesis	*accD*
	Proteins of unknown function	*ycf2*^c^*, ycf1*

^a^Gene containing one intron;

^b^Gene containing two introns;

^c^Two gene copies in the IRs;

^d^Pseudogene.

### Simple sequence repeat polymorphism analysis and hypervariable region in *Ficus* species

We detected five classes of SSRs in the chloroplast genomes of the 24 species of *Ficus*, including mono-, di-, tri-, tetra-, and pentanucleotide repeats. The total number of SSRs ranged from 67 (*F. sagittata*) to 89 (*F. microdictya*) (Supplementary Table 3). The most abundant SSRs were mononucleotides, accounting for 62.69% (*F. sagittata*) to 74.71% (*F. cyathistipula*), followed by dinucleotides (19.54% in *F. aurea* to 28.57% in *F. langkokensis*), trinucleotides (3.45% in *F. cyathistipula* to 7.50% in *F. cyrtophylla*), and tetranucleotides (1.12% in *F. microdictya* to 2.33% in *F. religiosa*), with only one pentanucleotide repeat found in *F. beipeiensis* (1.25%). The mononucleotide SSRs primarily consisted of A or T bases, while most dinucleotide SSRs consisted of AT or TA bases. Based on comparative analysis of SSRs at the subgenus level, mean number of SSRs ranged from 73.00 (subgenus *Synoecia*) to 86.50 (subgenus *Urostigma*) (Table 2). Fig species in the *Pharmacosycea* and *Urostigma* subgenera contained > 80 SSRs. In contrast, the total number of SSRs in the two *Synoecia* species was below 80 (Supplementary Table 3). Spearman rank correlation suggested that fig chloroplast sequence length and number of SSRs were strongly correlated (*R* = 0.48, *p* < 0.01) (Supplementary Figure 1).

**TABLE 2:** Simple sequence repeats (SSRs) types and mean number of chloroplast genomes of six *Ficus* subgenera.

SSR type	Repeat unit	*Urostigma*	*Pharmacosycea*	*Sycomorus*	*Ficus*	*Sycidium*	*Synoecia*
Mono	A/T	61.17	58.33	58.33	54.20	55.50	45.00
	C/G	1.17	1.00	0.83	0.90	0.50	2.00
Di	AG/CT	1.00	1.00	1.00	1.00	1.00	1.00
	AT/AT	17.33	19.67	17.17	19.00	16.00	19.50
Tri	AAG/CTT	1.00	1.00	1.00	1.00	1.00	1.00
	AAT/ATT	3.33	3.00	3.00	4.00	4.00	3.00
	ACT/AGT	0.00	0.33	0.00	0.00	0.00	0.00
	AGC/CTG	0.00	0.00	0.00	0.00	0.50	0.50
Tetra	AGAT/ATCT	1.00	1.00	1.00	1.00	1.00	1.00
	AAAC/GTTT	0.17	0.00	0.00	0.00	0.00	0.00
	AATT/AATT	0.33	0.00	0.00	0.00	0.00	0.00
	AAAT/ATTT	0.00	0.33	0.00	0.00	0.00	0.00
Penta	AAAAT/ATTTT	0.00	0.00	0.17	0.00	0.00	0.00
Total	/	86.50	85.66	82.50	81.10	79.50	73.00

**FIGURE 2:**
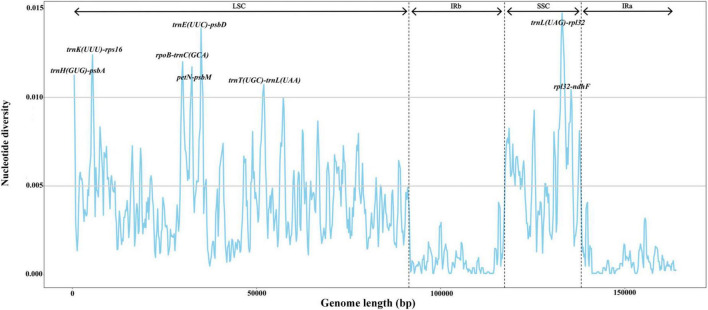
Nucleotide diversity (π) by sliding window analysis in the multiple alignments of 24 *Ficus* plastomes. Window length: 600 bp, step size: 200 bp. *X*-axis: the position of the midpoint of a window. *Y*-axis: the nucleotide diversity of each window.

To reveal hotspot regions for variability among fig chloroplasts, we used the mVISTA online tool to align and compare the plastome sequences, with *F. auriculata* (BN-FA10) used as a reference (Supplementary Figure 2). Results suggested that the *Ficus* chloroplast genomes were highly conserved. However, hypervariable regions were detected in some intronic and intergenic regions, including *rpl2-trnH(GUG)*, *trnH(GUG)-psbA*, *trnK(UUU)-rps16*, *rps16-trnQ(UUG)*, *rpoB-trnC(GCA)*, *petN-psbM*, *trnE(UUC)-trnT(GGU)*, *trnT(GGU)-psbD*, *psbZ-trnG(GCC)*, *trnT(UGU)-trnL(UAA)*, *trnL(UAG)-rpl32*, and *rpl32-ndhF*. In contrast, only a few differences were detected in the protein-coding regions, i.e., in *ycf2*, *ycf1*, and *ndhF*. To further assess sequence divergence in the *Ficus* chloroplast genomes, we calculated the nucleotide diversity (π) in DnaSP, which ranged from 0.0 to 0.01468. We also observed eight highly variable regions (π > 0.010), including six in the LSC region [*trnH(GUG)-psbA*, *trnK(UUU)-rps16*, *rpoB-trnC(GCA)*, *petN-psbM*, *trnE(UUC)-psbD*, and *trnT(UGU)-trnL(UAA)*] and two in the SSC region [*trnL(UAG)-rpl32* and *rpl32-ndhF*] (Figure 2). All hotspot regions existed in non-coding regions. The π values in the IR region were all below 0.005.

### Phylogenomic analysis and divergence time estimation

**FIGURE 3:**
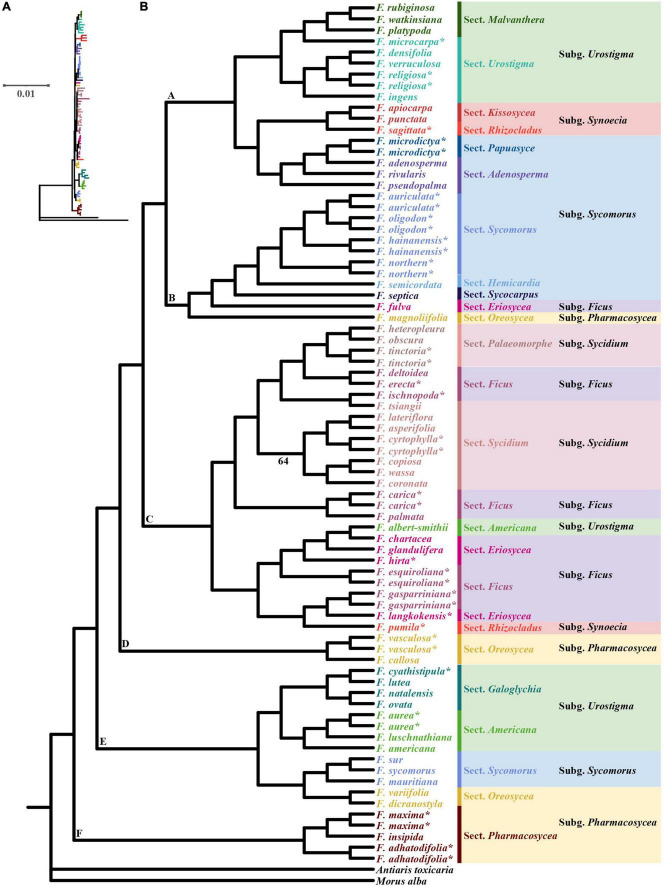
Maximum-likelihood (ML) phylogram **(A)** and cladogram **(B)** of 63 *Ficus* species inferred from whole chloroplast genome sequences. * Indicates the chloroplast was obtained in this study, the other 40 *Ficus* species were downloaded from Bruun-Lund et al. (2017). Only branches with weak support (bootstrap < 70%) are noted.

We constructed phylogenetic trees of the 63 species of *Ficus*. The ML and BI trees showed nearly identical topology, with high bootstrap support (BS) or posterior probabilities (Figure 3 and Supplementary Figure 3).

The ML tree showed section *Pharmacosycea* (clade F) as sister to all other figs with high bootstrap support (BS = 100%). After the basal split, a clade of two sections (*Galoglychia* and *Americana*) of *Urostigma*, three species of subgenus *Sycomorus* section *Sycomorus* and two species of subgenus *Pharmacosycea* section *Oreosycea* diverged (clade E, BS = 100%), and then was a clade comprising two species of subgenus *Pharmacosycea* sections *Oreosycea* (clade D, BS = 100%). Thereafter, all *Sycidium* and *Ficus* subgenera (except *F. fulva*, subgenus *Ficus* section *Eriosycea*) diverged, containing two species from subgenus *Urostigma* and *Synoecia* (clade C, BS = 100%). Clade B (BS = 99%) included the *F. auriculata* Lour. complex (*F. auriculata*, *F. oligodon*, *F. hainanensis*, and the new lineage, *Fn*), another two subgenus *Sycomorus* sections *Hemicardia* and *Sycocarpus*, as well as *F. fulva* and the last species of *Pharmacosycea* section *Oreosycea*. Clade A (BS = 97%) included two sections of *Urostigma* (*Malvanthera* and *Urostigma*), *Synoecia* (*Kissosycea* and *Rhizocladus*), and *Sycomorus* (*Papuasyce* and *Adenosperma*), respectively.

**FIGURE 4:**
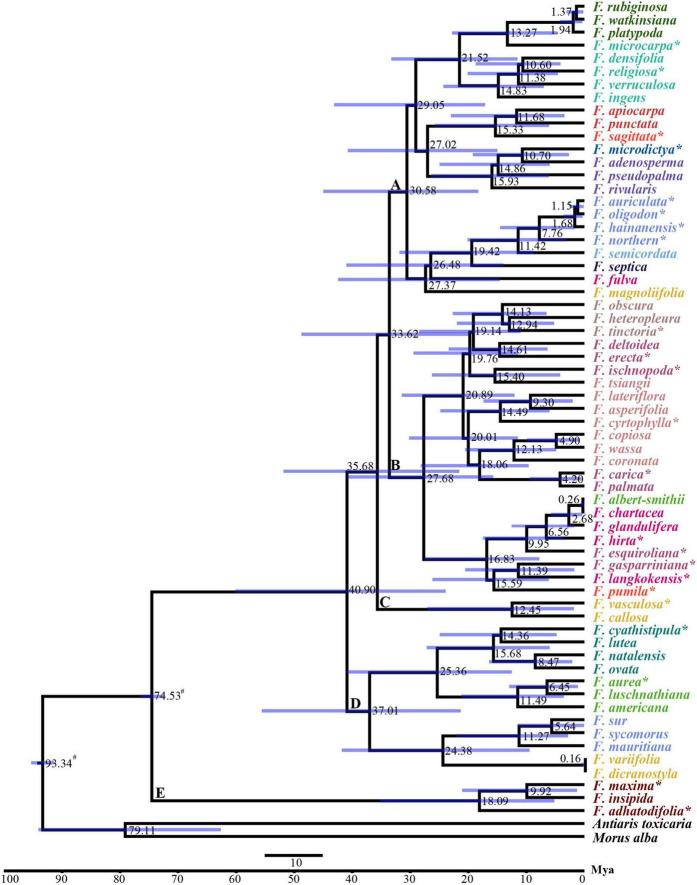
BEAST chronograms of the evolutionary history of *Ficus* using Yule model. The mean divergence time of the nodes was shown next to the nodes while the blue bars correspond to the 95% highest posterior density (HPD). ^#^ Indicates the calibration points, * indicates the chloroplast was obtained in this study, the other 40 *Ficus* species were downloaded from Bruun-Lund et al. (2017).

We estimated the divergence times of the 63 species of *Ficus* based on the BI tree. Both the Yule and Birth-Death models suggested that the species shared a common ancestor approximately 74.5 Mya [95% highest posterior density (HPD): 72.58–76.54 Mya] (Figure 4 and Supplementary Figure 4). The Yule model showed that *Ficus* diverged into all other clades around 40.90 Mya (95% HPD: 23.90–60.11 Mya). Divergence time between the two major clades (clades A and B) was estimated at 33.62 Mya (95% HPD: 19.96–48.75 Mya). Two species (*F. vasculosa* and *F. callosa*) in clade C diverged 12.45 Mya (95% HPD: 1.83–27.05 Mya) and species in clade D diverged 37.01 Mya (95% HPD: 21.32–55.58 Mya). For the clade containing *F. auriculata* Lour. Complex was dated to 7.76 Mya (95% HPD: 1.73–14.55 Mya) (Figure 4). Most divergence times estimated using the Birth-Death model were nearly identical (with slight variation) (Supplementary Figure 4).

**FIGURE 5:**
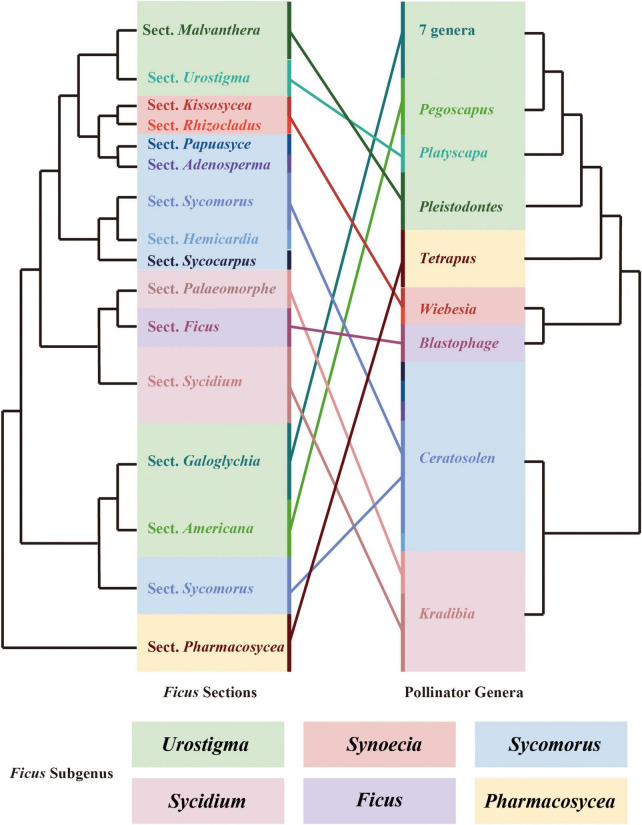
Cophylogenetic comparison of sections of *Ficus* and genera of pollinating wasps. Both phylogenetic trees were pruned. Colored boxes indicate the subgeneric classification of *Ficus*. Colored lines connect the sections of *Ficus* and their related genus of pollinators.

Co-phylogenetic comparison of figs and their pollinating wasps showed that the *Ficus* subgenera were associated with the fig wasp genera. However, we also observed one pollinating wasp genus that was related to more than one *Ficus* section (Figure 5).

### Selective pressure analysis of all plastome protein-coding genes in *Ficus*

**FIGURE 6:**
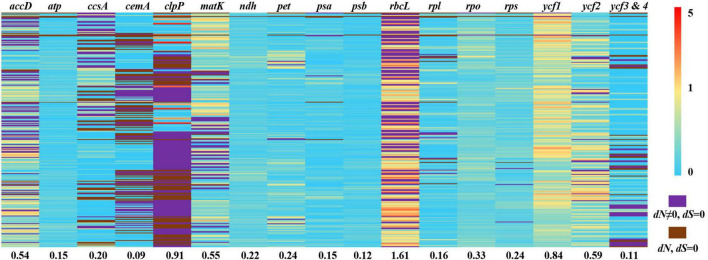
Pairwise non-synonymous rates (*dN*)/synonymous rates (*dS*) ratios in *Ficus*. This heatmap shows pairwise *dN*/*dS* ratios of 78 protein-coding genes from 24 *Ficus* species. The number under each gene dataset represented the mean *dN*/*dS* ratio. The order of species pairs showed in Supplementary Table 8.

We used branch, site, and branch-site models of the CODEML algorithm in EasyCodeML to analyze the likelihood of positive selection acting on *Ficus* protein-coding genes. After removing *F. microdictya*, *F. tinctoria*, and *F. cyrtophylla*, the ML tree topology was similar to the above phylogenetic tree (Figure 3 and Supplementary Figure 3) and could be divided into six clades (clades A–F) (Supplementary Figure 5). We selected clades A, B, C, D, E, and F as foreground branches, respectively. In the site model, no positively selected sites were detected (Supplementary Table 4). In the branch-site model, 15 positively selected gene datasets were obtained, but the *p*-values of the likelihood ratios were > 0.05, except for *ccsA* (Supplementary Tables 5, 6). In the branch model, M0 revealed that the *clpP* gene had a ω value > 1 (2.29950) (Supplementary Table 7). We also calculated the pairwise *dN*/*dS* ratios for the 17 gene datasets separately (Figure 6). Among the genes, the mean *dN*/*dS* ratio of *rbcL* was the highest (∼1.61), followed by *clpP* (∼0.91), and positive selection signals were detected in more than half of the pairwise results for both genes (*dN*/*dS* ratio > 1). The remaining gene datasets had mean *dN*/*dS* ratios varying from 0.09 (*cemA*) to 0.84 (*ycf1*). Overall, only three genes (*ccsA*, *clpP*, and *rbcL*) were identified as under positive selection, with all other 75 genes under purifying selection.

## Discussion

### Conserved plastome structure and divergence hotspots in *Ficus*

Here, we explored the structure and variation in chloroplast genomes covering all six subgenera of the large tropical genus *Ficus*. Specifically, we showed that the chloroplast genomes of the 24 *Ficus* species were extremely similar in structure, size, gene content, and order, thus suggesting high conservation. The variation in chloroplast genome size was only ∼0.7 kb. Previous studies have reported that variation in plastome size within a genus is associated with contraction and expansion of IR regions (Ravi et al., 2007). However, gene distribution in all IR boundary regions of the *Ficus* chloroplast genomes were identical, possibly leading to only minor differences in chloroplast size in the genus. Nevertheless, plastome size in figs may be affected by the number of SSRs. SSRs play essential roles in genome recombination and rearrangement and can be found throughout the genome (Vieira et al., 2016). SSRs exhibit a high rate of polymorphism and significant variation at the species level and are thus valuable markers for studying genetic diversity, population structure, and biogeography within and between fig species (Moe and Weiblen, 2011). We identified 67–89 SSRs in the complete chloroplast genomes of the 24 *Ficus* species, with varying number among the six subgenera. We also demonstrated that *Ficus* plastome size increased linearly with increasing SSRs.

Gene number and distribution were conserved among almost all 24 *Ficus* species, except for *ndhF*, which was identified as a pseudogene in *F. microcarpa* and *F. vasculosa*. Of note, *ndhF* is a protein-coding gene of the NAD(P)H-dehydrogenase complex involved in the regulation of electron recycling within photosystem I (Krause, 2011; Wicke et al., 2011). The *ndhF* gene is frequently lost or pseudogenized in heterotrophic plants (Funk et al., 2007; Barrett et al., 2014; Lam et al., 2015), but has also been found pseudogenized or absent in several autotrophic plants, such as *Mikania cordata* (Asteraceae), *Pinus abies* (Pinaceae), and *Ephedra equisetina* (Ephedraceae) (Braukmann et al., 2009; Su et al., 2018; Strand et al., 2019). The pseudogenization of *ndhF* in *F. microcarpa* and *F. vasculosa* is due to a single-base deletion resulting in a premature stop codon. Previous studies have suggested that a functional copy of the gene may have been transferred to the nucleus in this situation (Wakasugi et al., 1994; Sugiura et al., 2003; Su et al., 2018). Further studies on the pseudogenization of *ndhF* may contribute to our understanding of *Ficus* evolution and adaptation.

Although the chloroplast genomes were highly conserved among the 24 *Ficus* species, we identified eight divergence hotspot regions [*trnH(GUG)-psbA*, *trnK(UUU)-rps16*, *rpoB-trnC(GCA)*, *petN-psbM*, *trnE(UUC)-psbD*, *trnT(UGU)-trnL(UAA)*, *trnL(UAG)-rpl32*, and *rpl32-ndhF*] based on mVISTA and sliding window analysis. All regions were found in the single-copy and intergenic regions. As observed in other angiosperms, the IR and coding regions exhibited lower levels of divergence than the single-copy and non-coding regions (Lu et al., 2016; Yin et al., 2018; Wu et al., 2021). Divergence hotpots in chloroplast genomes have been widely utilized for delimitation of closely related species of plants (Bi et al., 2018; Dong et al., 2021). Thus, we propose that the eight highly variable regions identified here may serve as DNA barcodes in *Ficus* and may be useful for studies on intraspecific phylogeography.

### Phylogenetic analysis and divergence time in *Ficus*

Compared with earlier research on *Ficus* plastomes sampled from Europe and America (Bruun-Lund et al., 2017), we added 11 new Asian *Ficus* species and reconstructed the phylogenetic tree based on 63 chloroplasts, which showed highly consistent phylogeny with previous research. The use of additional samples can help resolve the complex *Ficus* phylogeny with greater confidence (Cruaud et al., 2012). For example, Bruun-Lund et al. (2017) divided species in the subgenus *Sycidium* section *Sycidium* into three clades dispersed within the subgenus *Ficus* and subgenus *Sycidium* section *Palaeomorphe*, whereas our ML tree recovered the species as a clade (BS = 64%, except *F. tsiangii*). However, our chloroplast phylogenetic tree did not support any of the six traditionally recognized subgenera of *Ficus* as monophyletic. Our placement of section *Pharmacosycea* (*F. maxima* and *F. adhatodifolia*) is consistent with previous study based on a nuclear genomic data, providing strong support that this section is sister to all other figs (Bruun-Lund et al., 2017; Wang et al., 2021). However, a recent study used genome-wide RAD loci and morphological features to infer the phylogenetic relationships in *Ficus* revealed that long-branch attraction led to section *Pharmacosycea* was the basal clade (Rasplus et al., 2021). Their results also strongly support the subgenera *Sycidium*, *Sycomorus*, and *Urostigma* as monophyletic clades, and suggest that the polyphyletic nature of these chloroplast genome-based clades may be due to heterogeneity (Rasplus et al., 2021). Thus, more research is needed to determine the reasons for nuclear and plastid discordance in figs, as it is unrealistic to rely on single genomes or small sample sizes to resolve these questions. Our phylogenetic results provided strong support for the monophyly of the *F. auriculata* Lour. complex. The taxa comprising this complex are sympatric in southeastern and southern China, and subtropical and tropical regions of Southeast Asia (Corner, 1978; Berg, 2007). Among putative species, *F. auriculata*, *F. oligodon*, and *F. hainanensis* are thought to have undergone natural hybridization and introgression, as based on SSR markers (Wei et al., 2014). Additionally, these species share pollinators, which may be the underlying mode of introgression (Wang et al., 2016). In this study, we identified a new genetic lineage (*Fn*), initially determined using nuclear microsatellite data (Wei et al., 2014). Based on chloroplast genomes, we used two models to estimate divergence times, both of which suggested that figs originated in the Late Cretaceous, approximately 74.5 Mya, then underwent rapid diversification in the early to middle Miocene (21.52–11.68 Mya). This rapid speciation coincided with global warming during the early Miocene and global maximum temperatures during the Miocene Climatic Optimum (MCO; 17–14.5 Mya) (Riera et al., 2021). Rapid speciation in the Miocene detected using the chloroplast genome is similar to that observed using nuclear genomes (Cruaud et al., 2012; Wang et al., 2021).

Co-evolution is defined as an evolutionary process whereby the traits of one species influence the evolution of the traits of another species, and vice versa (Janzen, 1980). The long-term co-evolution of figs and fig wasps has produced several adaptive traits. For example, the head, mandible, antenna, and ovipositor morphology and structure of pollinating wasps are co-adapted to fig traits such as the size of the enclosed inflorescences, aperture of the bracts, length of the styles, morphology of the stigma, and phenology of inflorescences (Ramirez, 1974; Janzen, 1979; Weiblen, 2002). Figs also release specific volatile organic compounds during the receptive phase to attract obligate pollinators and non-pollinating wasps (Hossaert-Mckey et al., 2010; Chen et al., 2016). These co-adaptation traits may have promoted the co-diversification of *Ficus* and pollinator wasps over the past 75 million years. In this study, we explored the co-phylogeny of *Ficus* and their pollinators above the species level due to the limited samples and genomic data. Our results supported high co-evolution between *Ficus* sections and fig wasp genera, as reported in previous morphological (Sonibare et al., 2004) and molecular studies (Machado et al., 2005; Cruaud et al., 2012). In general, closely related fig wasps pollinate closely related fig trees. However, we identified several *Ficus* sections associated with one pollinating wasp genus. For example, several sections of the subgenus *Sycomorus* were associated with the *Ceratosolen* genus in *Ficus*, and the genus *Kradibia* pollinated sections *Palaeomorphe* and *Sycidium* in the subgenus *Sycidium*. Thus, at the species level, the general view that one fig wasp species is associated with a particular host fig is challenged by several cases of breakdown of the one-to-one rule in fig-fig wasp mutualism. At present, however, two-thirds of pollinating wasps remain to be identified and a robust fig wasp phylogeny is still lacking, thereby hindering resolution of the incongruencies among fig-fig wasp co-phylogeny. Therefore, further studies with additional *Ficus* and pollinator wasp samples are needed with advanced genomic markers to construct the fig-fig wasp co-phylogeny (Rasplus et al., 2021).

### Adaptive evolution in *Ficus*

Figs are present in tropical and subtropical regions worldwide and within heterogeneous ecological niches (Harrison, 2005). This suggests the occurrence of adaptive radiation, which may have left a selection footprint on the chloroplast genome. Our results indicated that chloroplast genes *clpP*, *rbcL*, and *ccsA* were subjected to positive selection, indicating that they may have contributed to environmental adaptation in *Ficus*. The *clpP* gene, which encodes *clpP* protease, is also positively selected in several angiosperm lineages, such as *Paphiopedilum* (Orchidaceae) (Guo et al., 2021), *Acacia* (Fabaceae) (Dugas et al., 2015), and *Bupleurum* (Apiaceae) (Huang et al., 2021b), and shows hypervariability in Amaryllidaceae and Papilionoideae (Liu et al., 2022; Moghaddam et al., 2022), suggesting it may have accelerated substitution rates in many angiosperms. Functional studies have also indicated that *clpP* protease degrades or restores damaged proteins (Wicke et al., 2011), and is important for changes in plant development in response to stress (Erixon and Oxelman, 2008). Hence, positive selection in this gene may help explain the radiation of *Ficus* into diverse ecological niches. The *rbcL* gene, which is a photosynthesis-related gene encoding the large subunit of ribulose-1,5-bisphosphate carboxylase/oxygenase (RuBisCO), is commonly under positive selection in terrestrial plants but not in algae (Kapralov and Filatov, 2007; Li et al., 2022), possibly due to more unstable thermal regimes on land (Kapralov and Filatov, 2007). For example, *rbcL* is under positive selection in shade-tolerant *Oryza* species, probably in response to adaptive evolution associated with sunlight and thermal conditions (Gao et al., 2019b). In this study, we collected *Ficus* from habitats with diverse sunlight exposure, and thus *rbcL* may be involved in adaptation to different light intensities and thermal habitats in this genus. The *ccsA* (*ycf5*) gene encodes a protein required for cytochrome biogenesis that mediates the attachment of heme to c-type cytochromes (Xie and Merchant, 1996). Here, this gene was positively selected in clade E (*F. aurea* and *F. cyathistipula*). Previous research has reported that *ccsA* is under positive selection in orchids, including epiphytic species (Dong et al., 2018). In our analysis, *F. aurea* and *F. cyathistipula* were epiphytes, and thus *ccsA* may play an important role in the adaptation of epiphytes to special habitats. Additionally, *F. cyathistipula* was the only species distributed in the Afrotropics, and positive selection on *ccsA* may be related to specific environmental conditions, such as light intensity, moisture, and/or temperature. In summary, these three positively selected genes may contribute to the adaptation of different *Ficus* life-forms to diverse environments and may serve as candidate genes for further research on the mechanisms of adaptive evolution of the genus.

We also found that the remaining 75 genes in the 24 chloroplast genomes of *Ficus* were under purifying selection, suggesting low synonymous and/or non-synonymous DNA substitution. These results are similar to previous findings showing that most chloroplast genes are under purifying selection in Rosales (Souza et al., 2020; Wang et al., 2020; Yang et al., 2021a) and angiosperm species (Henriquez et al., 2020; Yang et al., 2020; Huang et al., 2021b), with a highly conserved evolutionary history. Purifying selection helps stop mutations from becoming fixed (Wu et al., 2020), thus eliminating deleterious mutations and retaining conserved function in genes (Huang et al., 2021b). In *Ficus*, purifying selection likely explains the high levels of interspecific conservation among chloroplast genomes.

## Conclusion

Our study supported the conserved structure of *Ficus* chloroplast genomes and revealed eight mutational hotspot regions, which may be utilized as high-resolution DNA markers for figs in future phylogenetic and phylogeographic studies. We detected positive selection in three genes (*clpP*, *rbcL*, and *ccsA*), which may be linked to adaptive evolution in *Ficus*. Phylogenetic analysis showed that none of the six traditionally recognized subgenera of *Ficus* were monophyletic. Thus, further research and additional taxonomic sampling are needed to explain the potential discordance with morphology and nuclear genomes. Overall, our study provides a new framework for an improved understanding of species delimitation, genome evolution, and phylogenetic relationships in *Ficus*.

## Data availability statement

The 13 new sequencing data presented in the study are deposited in the NCBI. The link to the repository and accession numbers can be found below: https://www.ncbi.nlm.nih.gov/genbank, ON711000–ON711012.

## Author contributions

JG and Y-QP conceived and designed the study. JG and W-YL collected the samples. Z-RZ and XY analyzed the data. JG, Z-RZ, and XY wrote the manuscript. Y-QP gave suggestions during the manuscript writing. All authors contributed to the article and approved the submitted version.

## Footnotes

1
https://bigd.big.ac.cn/gsa/

2
https://chlorobox.mpimp-golm.mpg.de/OGDraw.html

3
https://genome.lbl.gov/vista/mvista/submit.shtml

4
https://webblast.ipk-gatersleben.de/misa/
