# Supplementary figures and images for: Comparative chloroplast genome analysis of Ficus (Moraceae): Insight into adaptive evolution and mutational hotspot regions
Source: Front Plant Sci. 2022 Sep 15;13:965335. doi: 10.3389/fpls.2022.965335 (PMC9521400; doi:10.3389/fpls.2022.965335)

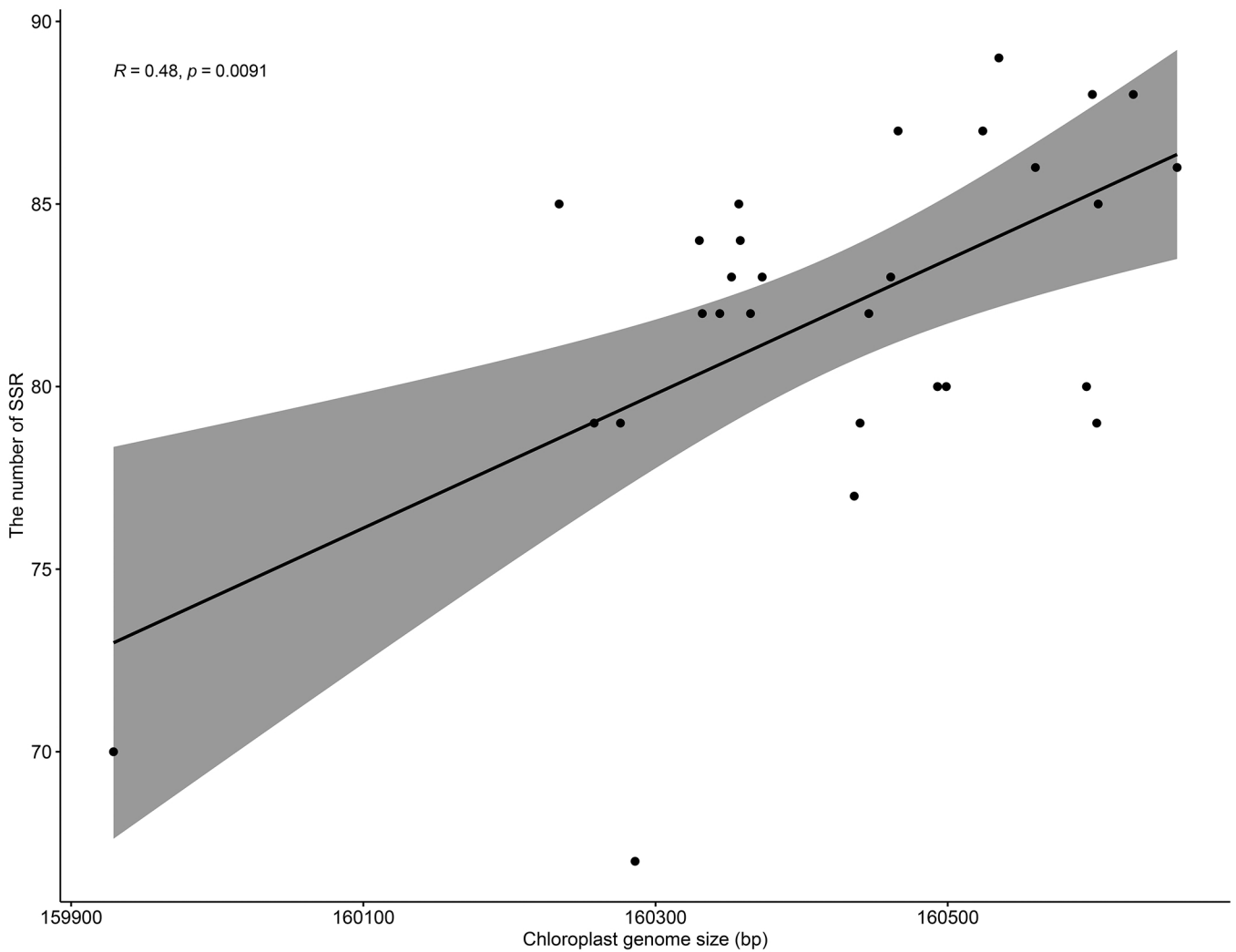

Supplement: Supplementary file 1 [file Data_Sheet_1.ZIP › Supplementary Material/Supplementary Figure 1.pdf]

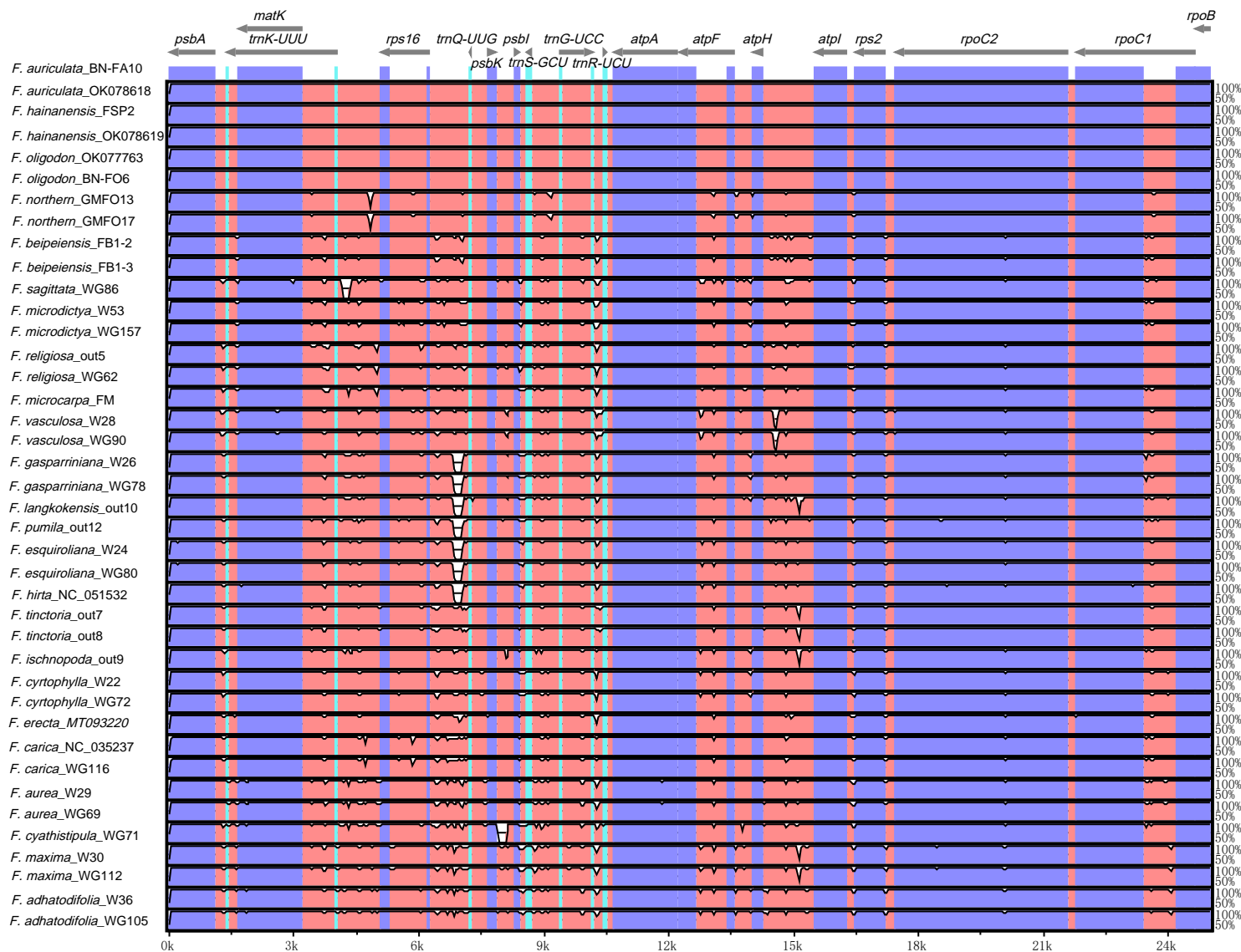

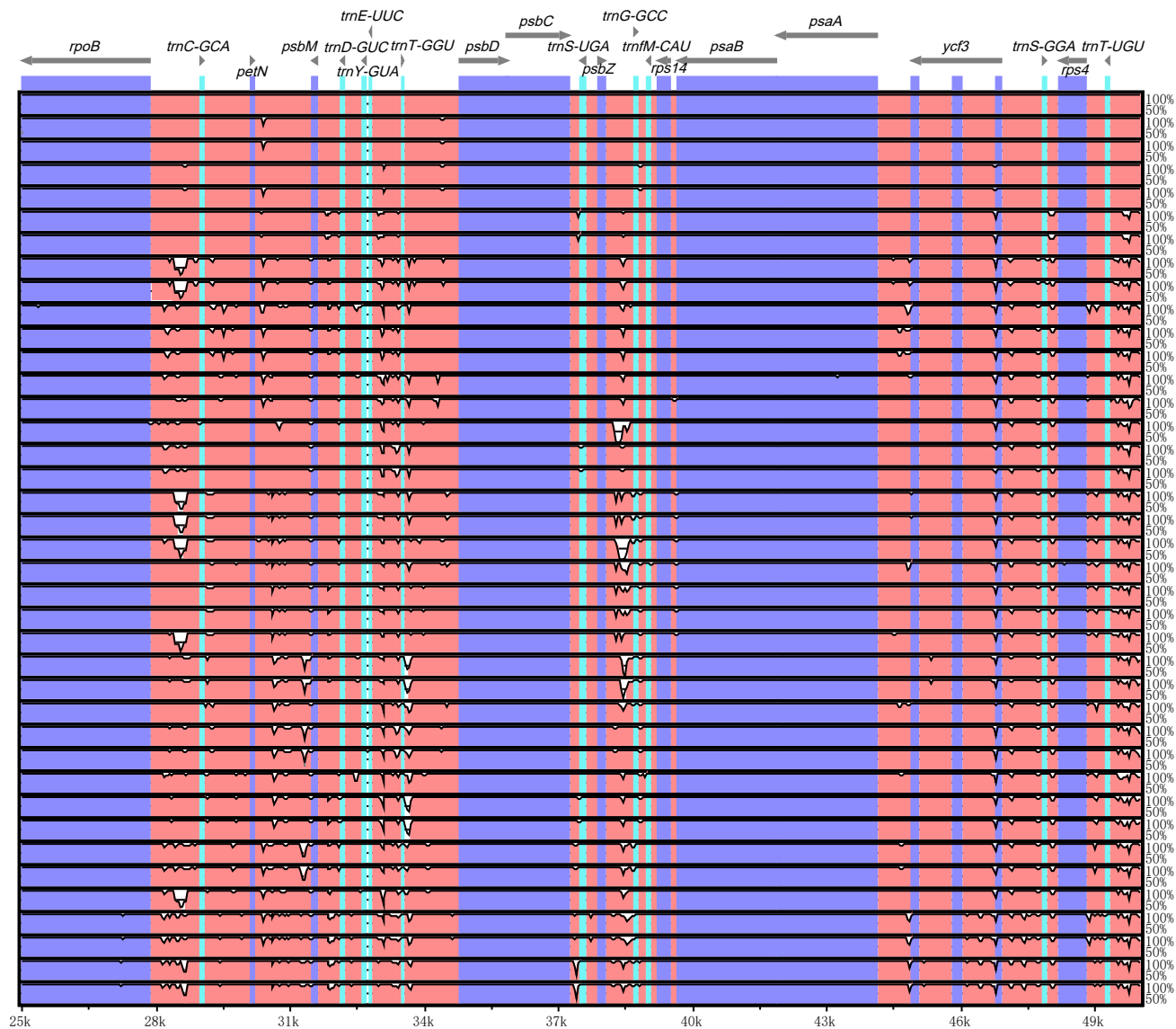

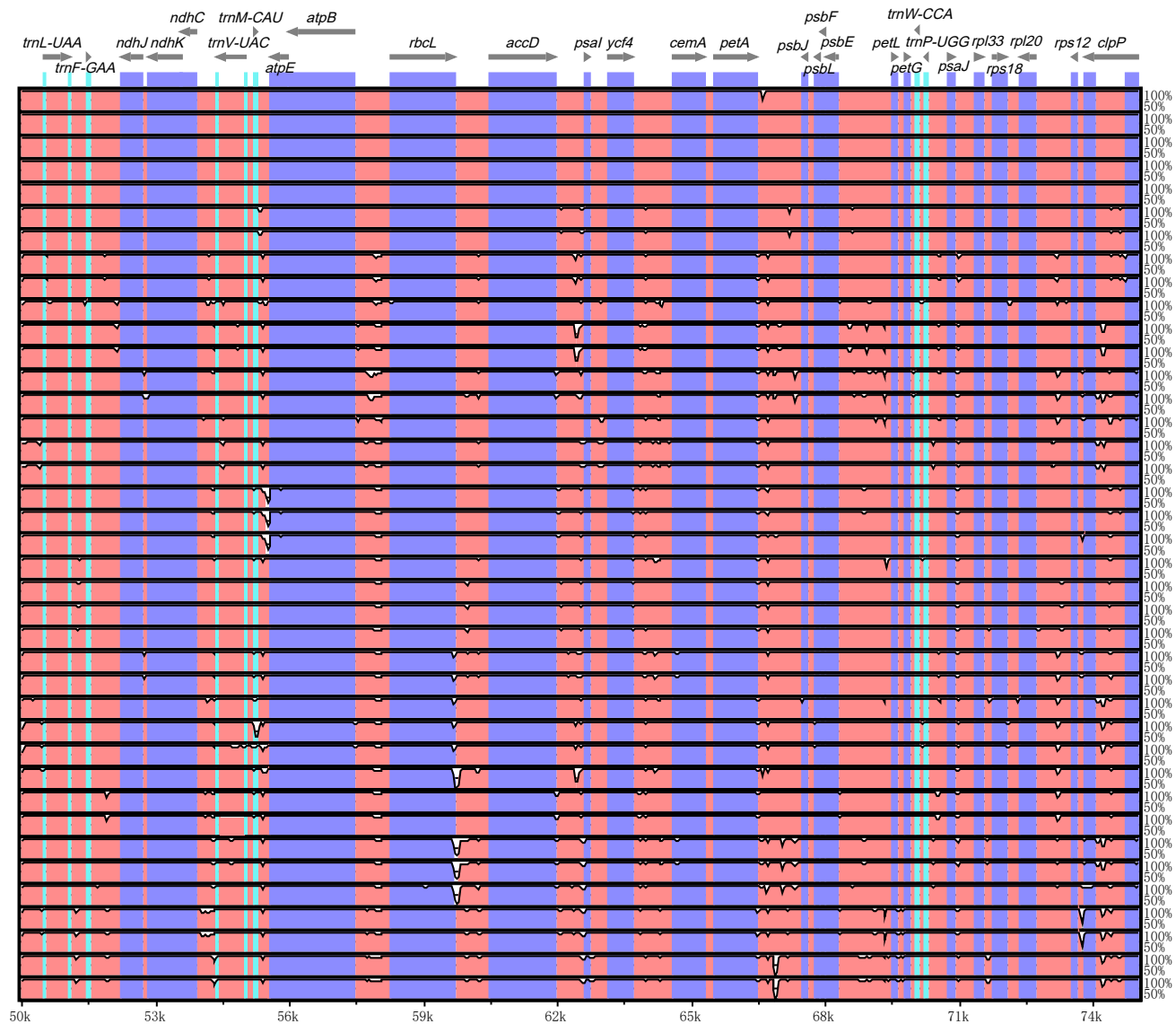

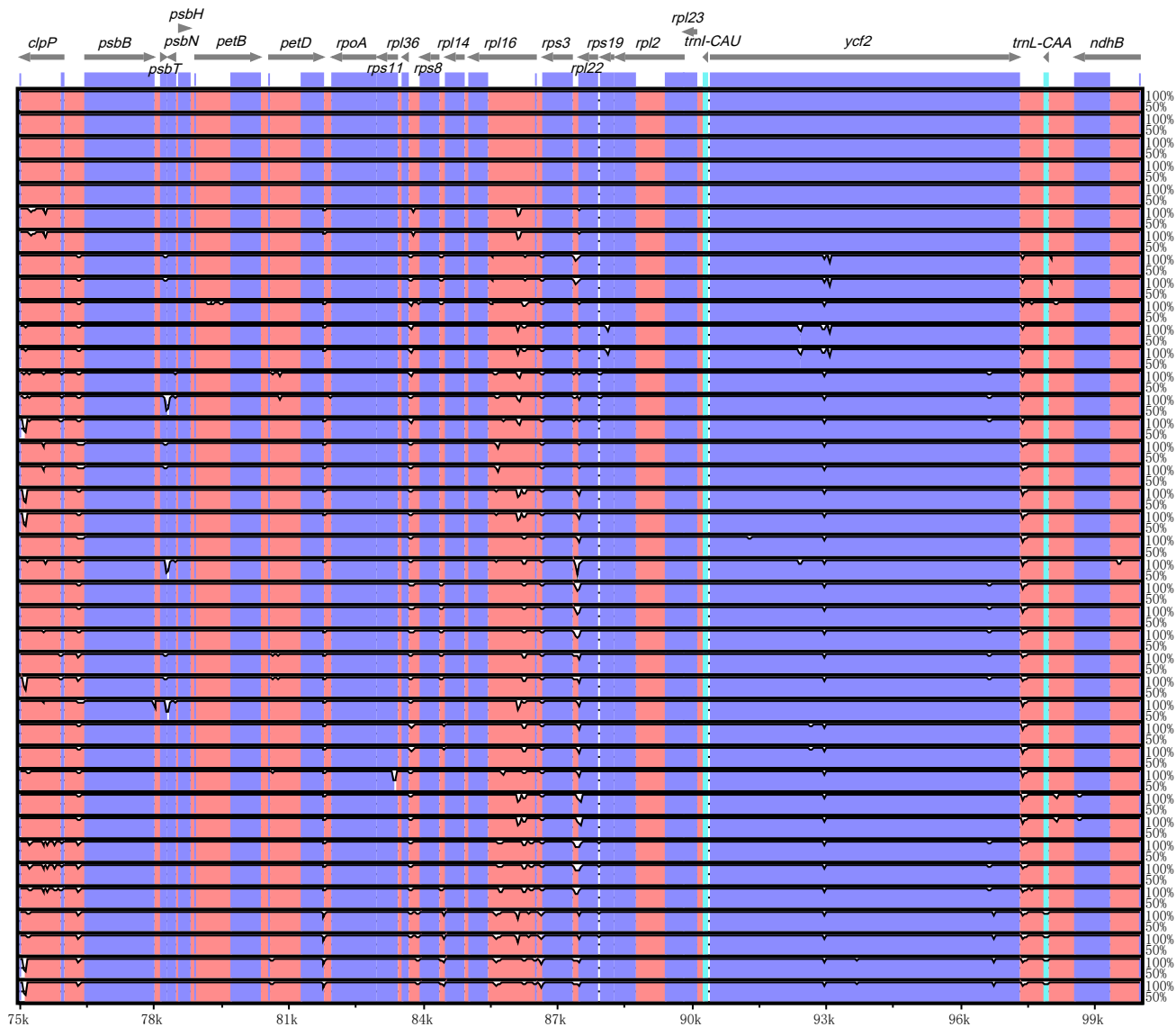

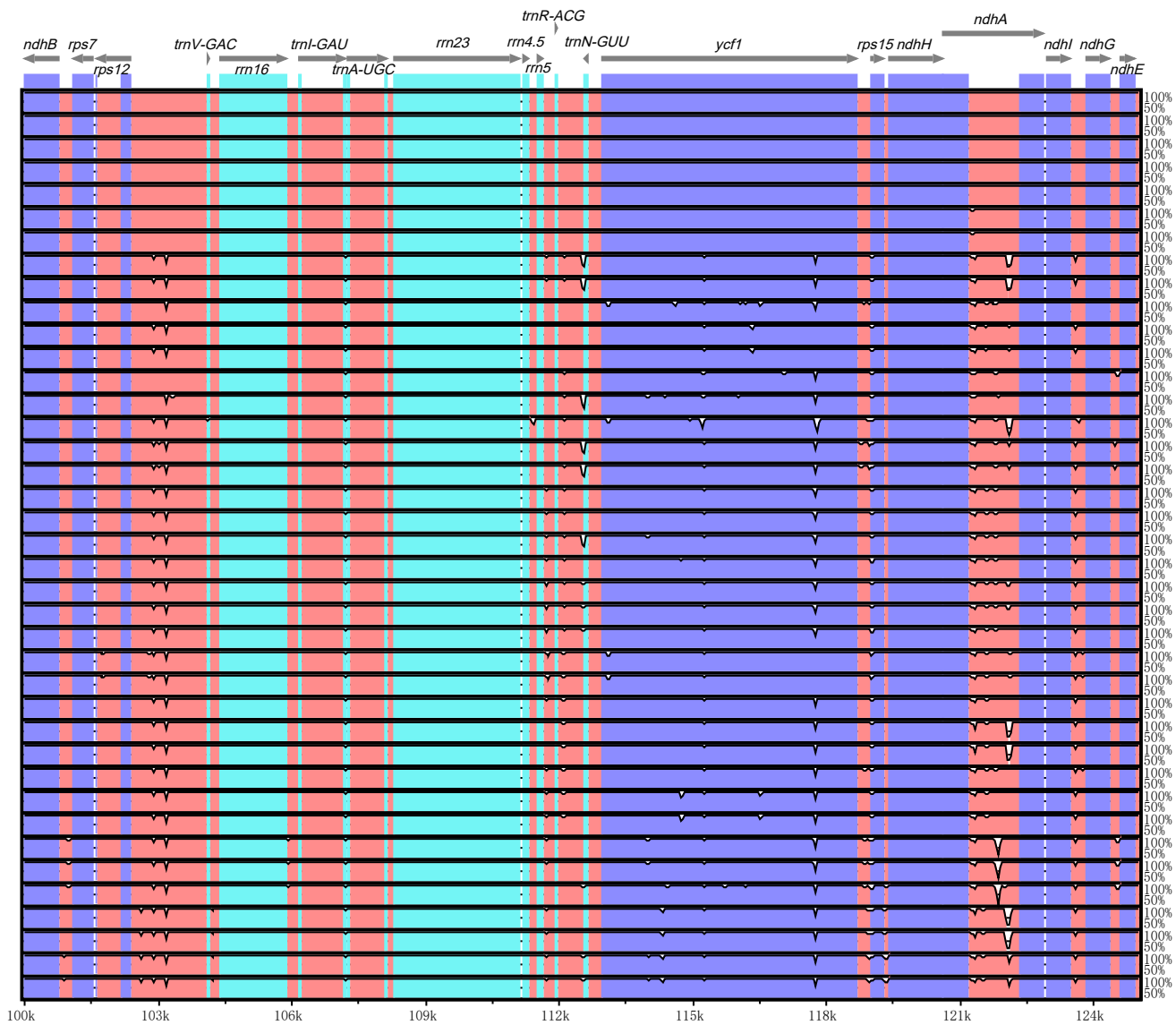

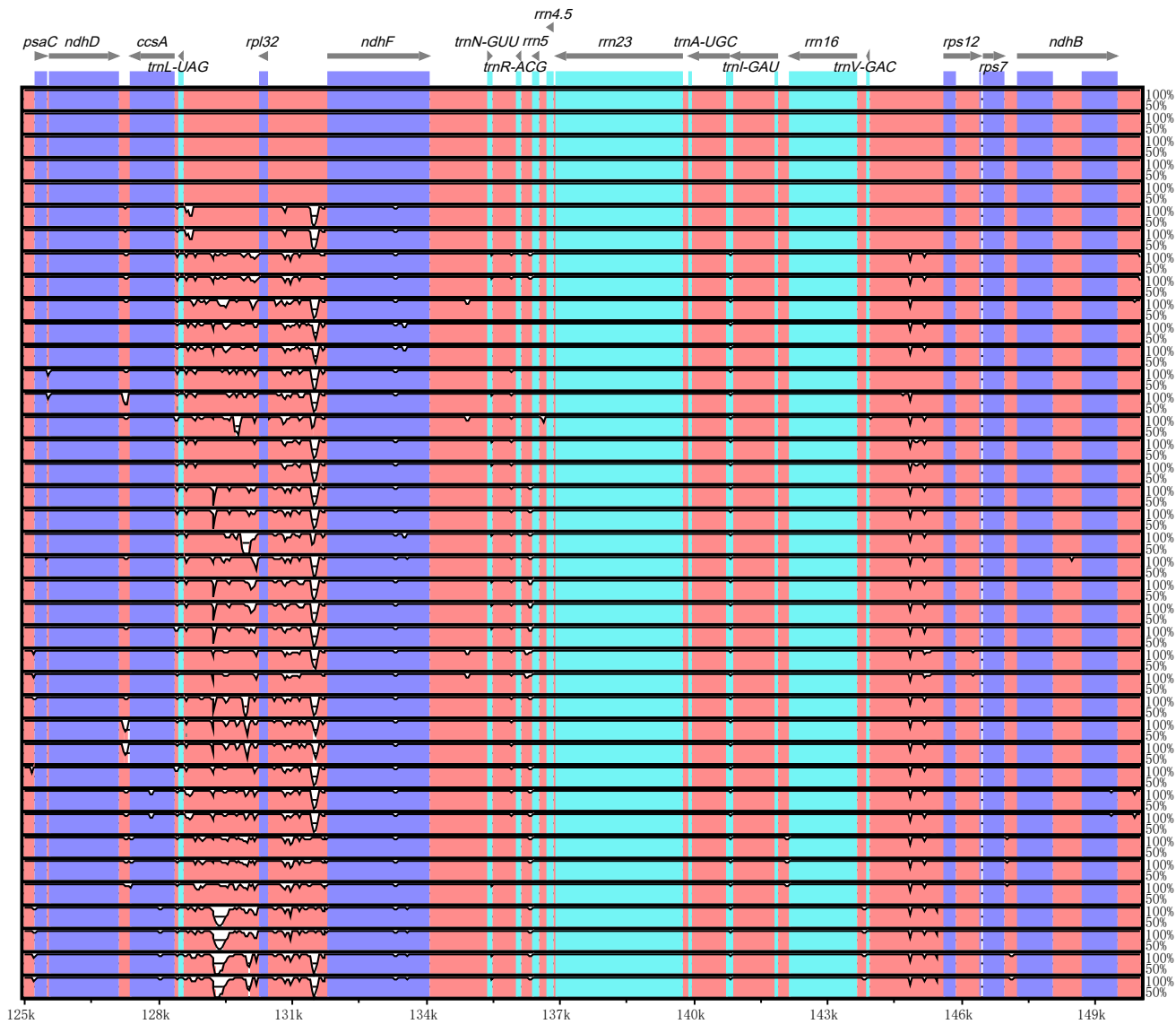

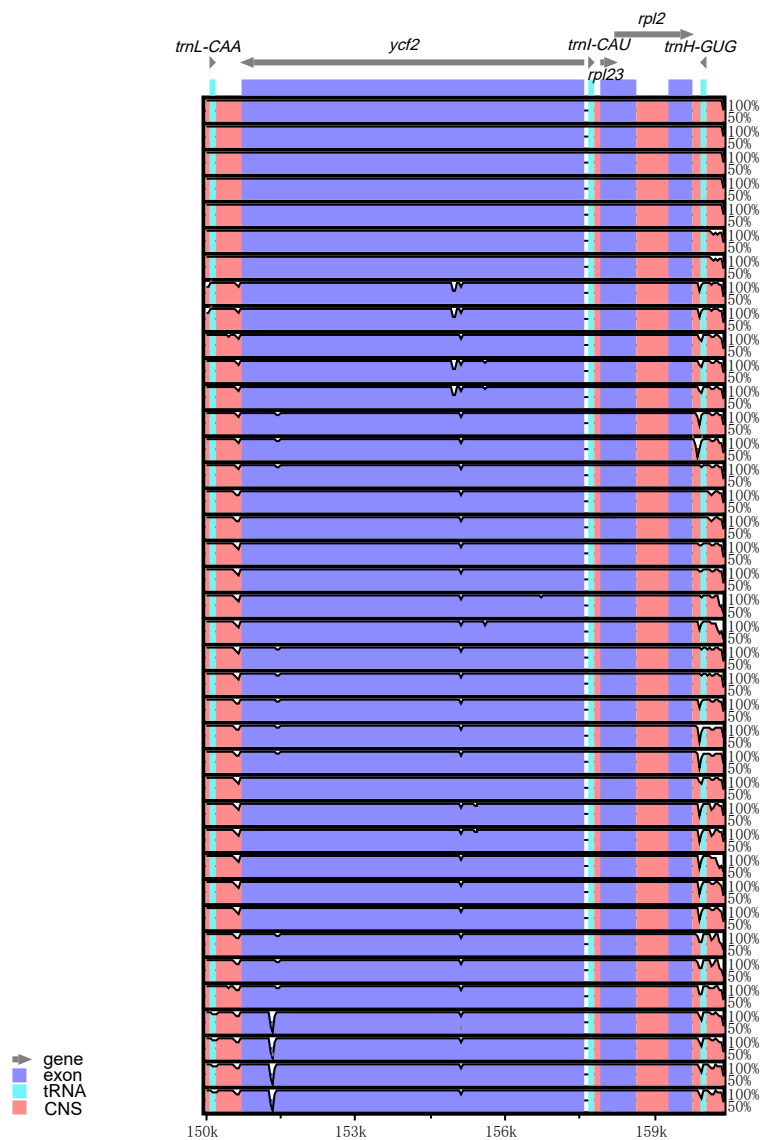

Supplement: Supplementary file 1 [file Data_Sheet_1.ZIP › Supplementary Material/Supplementary Figure 2.pdf]

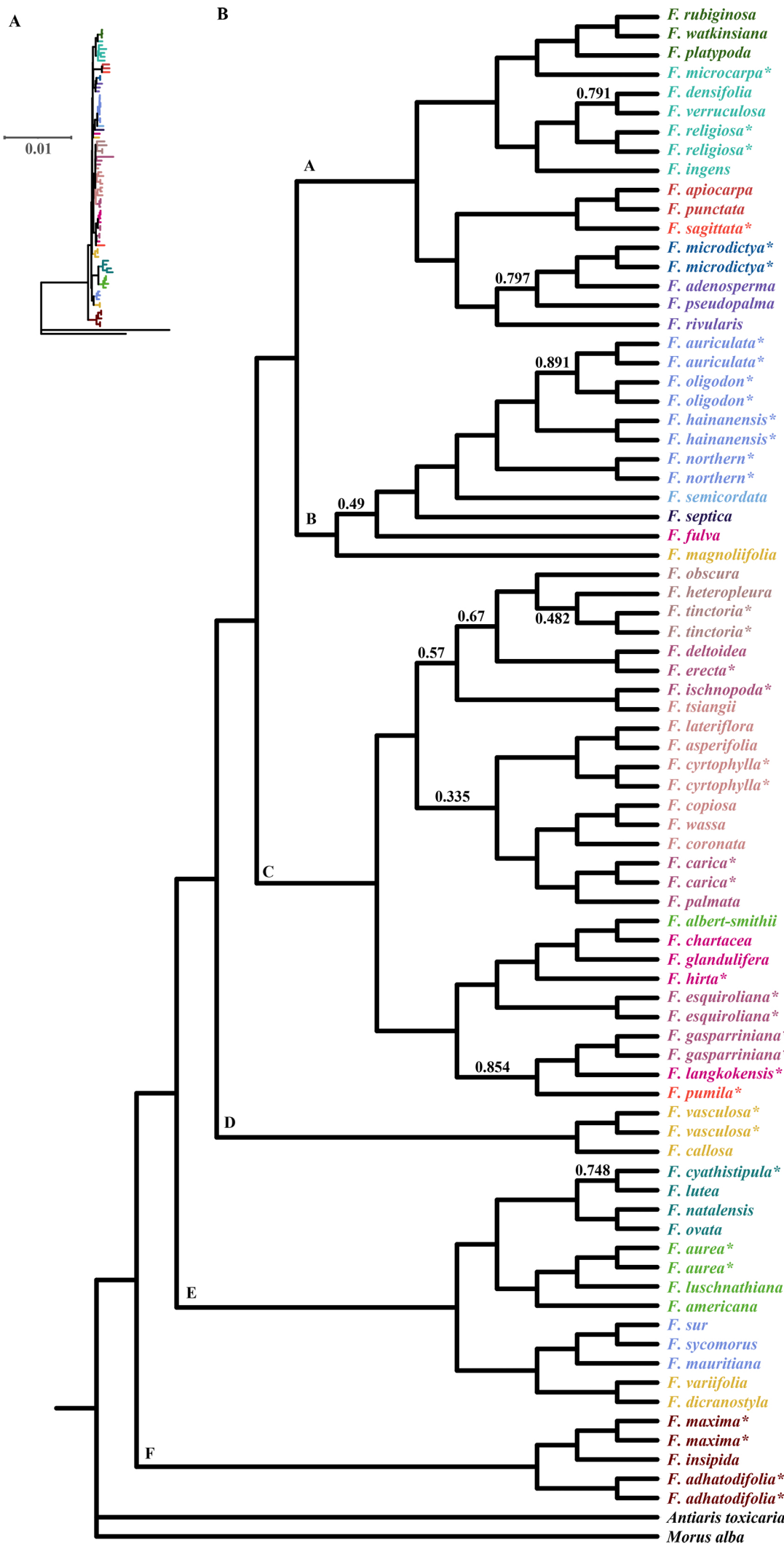

Supplement: Supplementary file 1 [file Data_Sheet_1.ZIP › Supplementary Material/Supplementary Figure 3.pdf]

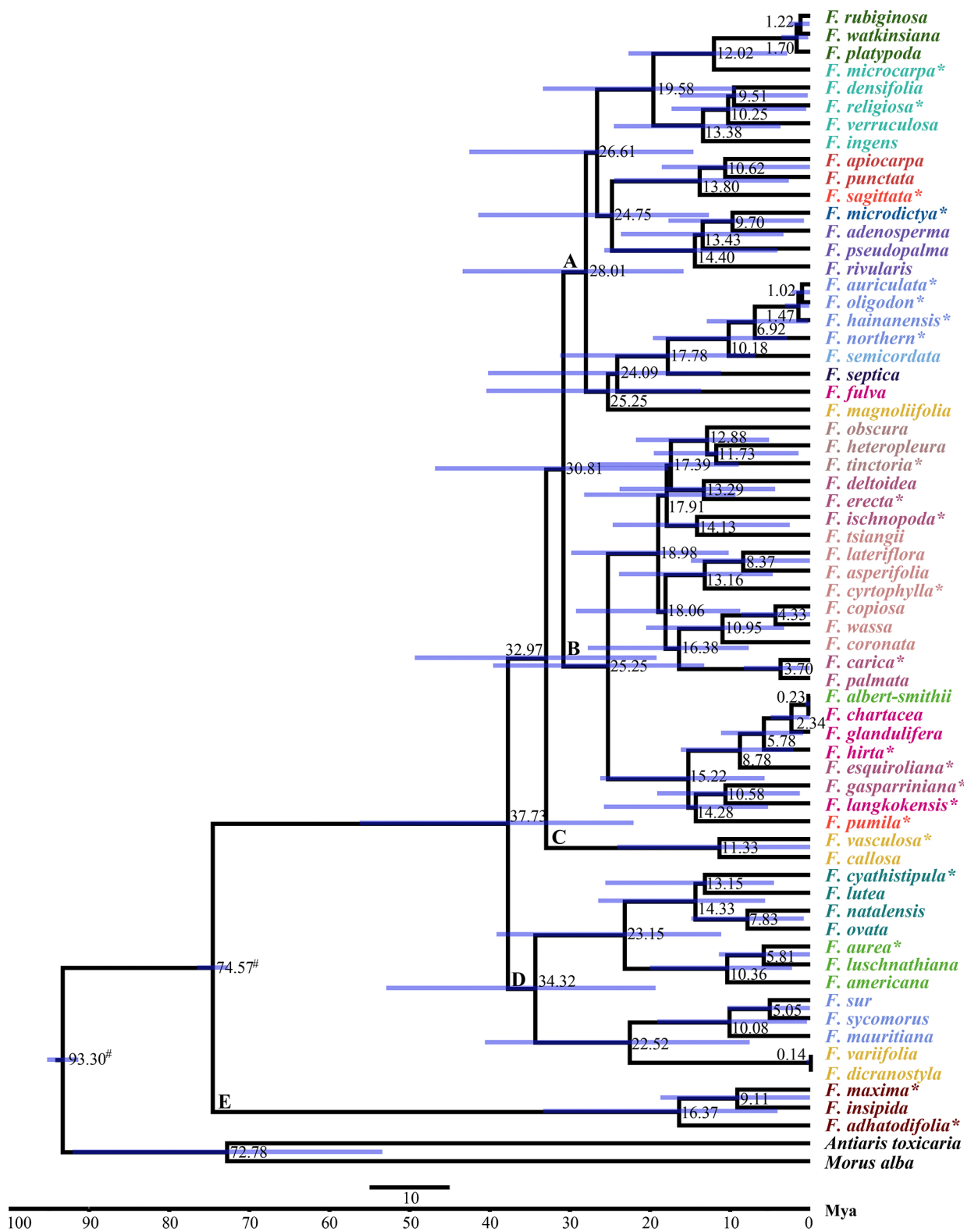

Supplement: Supplementary file 1 [file Data_Sheet_1.ZIP › Supplementary Material/Supplementary Figure 4.pdf]

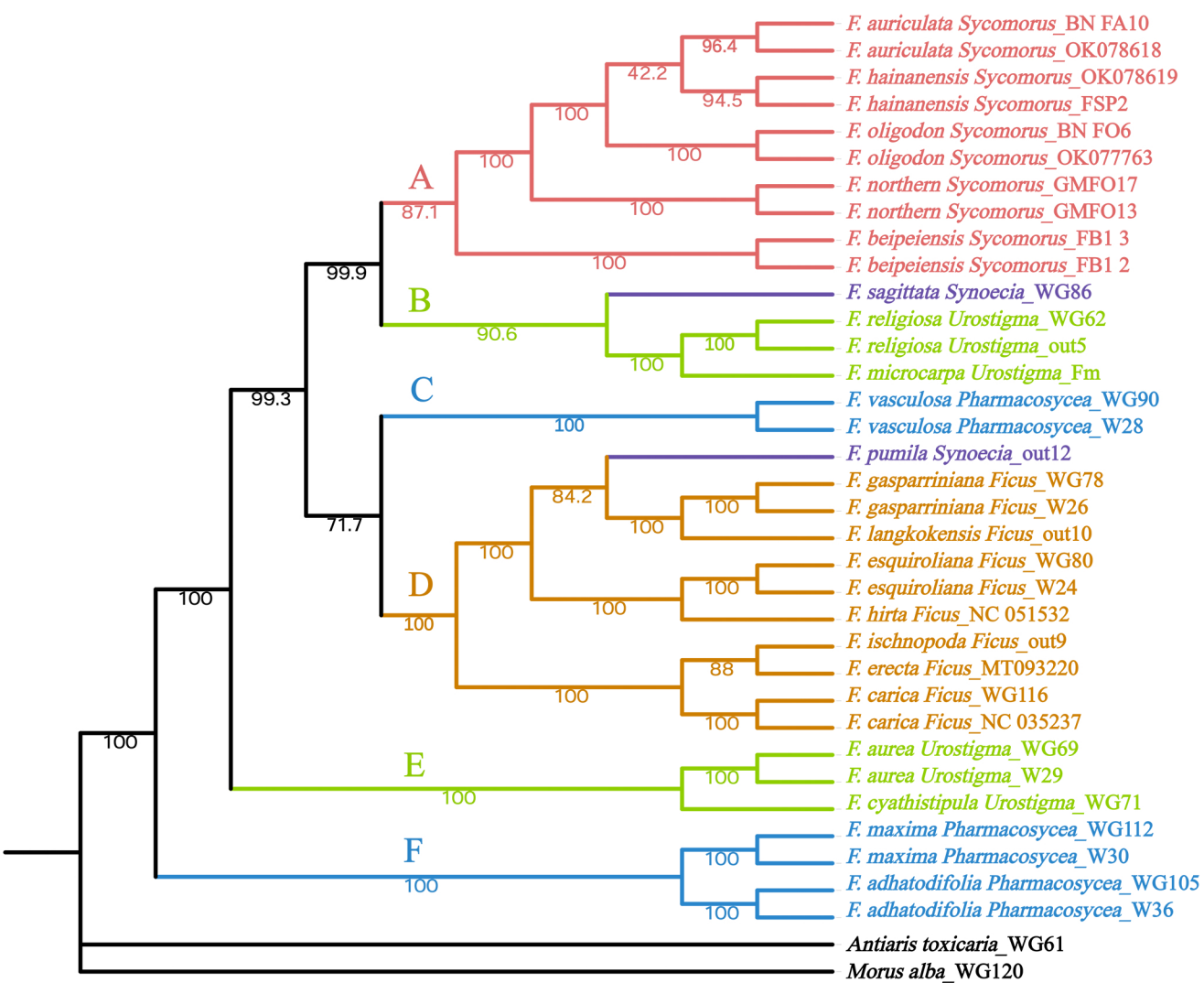

Supplement: Supplementary file 1 [file Data_Sheet_1.ZIP › Supplementary Material/Supplementary Figure 5.pdf]
