# Supplementary material for: Comparative chloroplast genome analysis of Ficus (Moraceae): Insight into adaptive evolution and mutational hotspot regions
Source: Front Plant Sci. 2022 Sep 15;13:965335. doi: 10.3389/fpls.2022.965335 (PMC9521400; doi:10.3389/fpls.2022.965335)
Supplement: Supplementary file 2 [file Table_1.docx]

Supplementary Material

# Supplementary Figures and Tables

## Supplementary Figures

**Supplementary Figure 1.** The correlation between *Ficus* chloroplast genome sizes and the number of Simple sequence repeats (SSRs). Spearman rank correlation=0.48, and *p*-value <0.01.

**Supplementary Figure 2.** Global alignment of *Ficus* Plastomes using mVISTA. X-axis: the coordinate in the chloroplast genome. Y-axis: the level of variation (between 50 and 100%).

**Supplementary Figure 3.** Bayesian inference (BI) phylogram **(A)** and cladogram **(B)** of 63 *Ficus* species inferred from whole chloroplast genome sequences. * indicates the chloroplast was obtained in this study, the other 40 *Ficus* species were downloaded from Bruun-Lund et al. (2017). Only branches with weak support (posterior probabilities < 0.95) are noted.

**Supplementary Figure 4.** BEAST chronograms of the evolutionary history of *Ficus* using Birth-Death model. The mean divergence time of the nodes was shown next to the nodes while the blue bars correspond to the 95% highest posterior density (HPD). ^#^ indicates the calibration points, * indicates the chloroplast was obtained in this study, the other 40 *Ficus* species were downloaded from Bruun-Lund et al. (2017).

**Supplementary Figure 5.** Maximum-likelihood (ML) Phylogenetic tree of selective pressure analysis of 78 protein-coding genes from *Ficus*. The tree was divided into 6 clades, defined as A, B, C, D, E, and F.

## Supplementary Tables

**Supplementary Table 1.** Samples information. List of *Ficu****s*** Samples information studied here.

**Supplementary Table 2.** Summary features of the 24 *Ficus* species chloroplast genomes characterized.

**Supplementary Table 3.** Simple sequence repeats (SSRs) analyses of 24 *Ficus* species chloroplast genomes.

**Supplementary Table 4.** Selective pressure analysis of 17 protein-coding gene datasets in *Ficus* chloroplast genomes (Site model).

**Supplementary Table 5.** Selective pressure analysis of 17 protein-coding gene datasets in *Ficus* chloroplast genomes (Branch-site model).

**Supplementary Table 6.** Positive selection sites of four genes were detected by the branch-site model.

**Supplementary Table 7.** Selective pressure analysis of 17 protein-coding gene datasets in *Ficus* chloroplast genomes (Branch model).

**Supplementary Table 8.** The order of species pairs for pairwise nonsynonymous rates (*dN*)/synonymous rates (*dS*) ratios in *Ficus*.
